# Exploitation of epigenetic variation of crop wild relatives for crop improvement and agrobiodiversity preservation

**DOI:** 10.1007/s00122-022-04122-y

**Published:** 2022-06-09

**Authors:** Serena Varotto, Tamar Krugman, Riccardo Aiese Cigliano, Khalil Kashkush, Ankica Kondić-Špika, Fillipos A. Aravanopoulos, Monica Pradillo, Federica Consiglio, Riccardo Aversano, Ales Pecinka, Dragana Miladinović

**Affiliations:** 1 Department of Agronomy Animal Food Natural Resources and Environment, University of Padova, Viale dell’Università, 16 35020 Legnaro, Italy; 2 Institute of Evolution, University of Haifa, Abba Khoushy Ave 199, 3498838 Haifa, Israel; 3 Sequentia Biotech, SL, Carrer de València, 08009 Barcelona, Spain; 4 Department of Life Sciences, Ben-Gurion University, Beersheba, 84105 Israel; 5 Institute of Field and Vegetable Crops, Maksima Gorkog 30, 21000 Novi Sad, Serbia; 6 Faculty of Agriculture, Forest Science & Natural Environment, Aristotle University of Thessaloniki, Thessaloniki, GR54006 Greece; 7 Department of Genetics, Physiology and Microbiology, Faculty of Biology, Complutense University of Madrid, 28040 Madrid, Spain; 8 Institute of Biosciences and Bioresources, National Research Council (CNR), Via Università 133, 80055 Portici, Italy; 9 Department of Agricultural Sciences, University of Naples Federico II, Via Università 100, 80055 Portici, Italy; 10 Institute of Experimental Botany, Centre of the Region Haná for Biotechnological and Agricultural Research, Czech Acad Sci, Šlechtitelů 31, 779 00 Olomouc, Czech Republic

## Abstract

Crop wild relatives (CWRs) are recognized as the best potential source of traits for crop improvement. However, successful crop improvement using CWR relies on identifying variation in genes controlling desired traits in plant germplasms and subsequently incorporating them into cultivars. Epigenetic diversity may provide an additional layer of variation within CWR and can contribute novel epialleles for key traits for crop improvement. There is emerging evidence that epigenetic variants of functional and/or agronomic importance exist in CWR gene pools. This provides a rationale for the conservation of epigenotypes of interest, thus contributing to agrobiodiversity preservation through conservation and (epi)genetic monitoring. Concepts and techniques of classical and modern breeding should consider integrating recent progress in epigenetics, initially by identifying their association with phenotypic variations and then by assessing their heritability and stability in subsequent generations. New tools available for epigenomic analysis offer the opportunity to capture epigenetic variation and integrate it into advanced (epi)breeding programmes. Advances in -omics have provided new insights into the sources and inheritance of epigenetic variation and enabled the efficient introduction of epi-traits from CWR into crops using epigenetic molecular markers, such as epiQTLs.

## Introduction

Historically, crop domestication and improvement involving recurrent selection to increase the frequency of desirable yield traits led to a massive loss of genetic variation (Bevan et al. 2017).

Many molecular studies confirmed a higher level of diversity in crop wild relatives (CWRs) than in the cultivated species, indicating that CWR can be used as a source for new variability in breeding programmes (Kondić-Špika et al. 2016; Heywood 2011; Jones et al. 2013). Thus, CWRs are recognized today as the best potential source for crop improvement (Bevan et al. 2017; Huang et al. 2016). Moreover, the successful exploitation of CWRs in crop improvement relies on identifying genetic variation in genes controlling desired traits in plant germplasm and subsequently incorporating them into cultivars (Nass and Paterniani 2000; Klymiuk et al. 2019). Plant genetic resources (PGRs) play a crucial role in this scenario, by offering a vast reservoir of important novel interesting traits and allelic variants. In addition, PGRs can contribute to the utilization of new species suitable for organic production, sustainable agriculture, food diversity, and stability of agricultural production systems. The synthesis of both intra- and interspecific variations of wild and domesticated plants will allow for more precise utilization of PGRs in the future. This could be achieved through pre-breeding programmes and the establishment of core collections, which contain the genetic diversity of a species and its relatives with a minimum of redundancy (Brown 1989). These collections can be dynamic, with new genotypes being introduced and the old replaced, depending on the needs of the breeding programmes (Anđelković et al. 2020).

Genetic diversity hidden in CWR is commonly considered at different levels: genome, locus, or DNA sequence. However, various types of epigenetic diversity may provide additional layers of diversity within a species and can contribute novel epialleles for some key traits. Research into the epigenetic mechanisms and epigenetic diversity involved in plant adaptation can be of particular value in supporting near future agricultural production challenges. Recent progress in molecular biology and analysis of chromatin structure and function has highlighted the complexity of epigenetic regulation in plants and their association with the desired traits. These progresses also advance the hypothesis that epigenetic variation can be exploited for selection in crop improvement. Therefore, deepening our understanding into the epigenetic mechanisms and epigenetic diversity involved in plant adaptation can be valuable in supporting future agricultural production challenges. Thus, concepts and techniques of classical and modern breeding should consider integrating recent progress in epigenetics, initially by identifying their association with phenotypic variations and then by assessing their heritability and stability in subsequent generations (Spinger and Schmitz 2017). In particular, CWR growing in contrasting environments can be used to identify gene (epi)alleles, which were subjects of long-lasting natural selection and adaptation to climate variations. Genetic and epigenetic diversity studies in natural CWR populations can therefore be used to guide crop improvement for climate resilience. So far, CWR sources have been exploited mainly for the major genes controlling tolerance to biotic and abiotic stress and/or for mining of genetically (and phenotypically) complex traits. However, the identification of epialleles and their characterization still remains a significant challenge. The new tools available for epigenomic analyses now enable capturing epigenetic variation and integrating it into advanced epibreeding programmes. Many reviews recently published (Gallushi et al. 2017; Lamke and Bäurle 2017; Johannes and Schmitz 2019) have provided descriptions of the molecular mechanisms that contribute to epigenetic variations in plants. Here, we focus on the contribution of epigenetic and chromatin states to plant biodiversity from the perspective of CWR and their exploitation in epibreeding programmes. Understanding how epigenetics can contribute to diversity is also fundamental to all steps of conservation and utilization of PGR in plant breeding. We concentrate on two case species in which CWR epigenetic variation has recently been studied and has the potential to be exploited for genetic improvement. We then describe the tools available for the characterization of epigenetic variation in non-model plants. Finally, we discuss the advantages and limitations of using CWR epigenetic variation in crop improvement and agrobiodiversity preservation.

## Natural epigenetic variation

### Environmental-triggered epigenetic variation

**Fig. 1:**
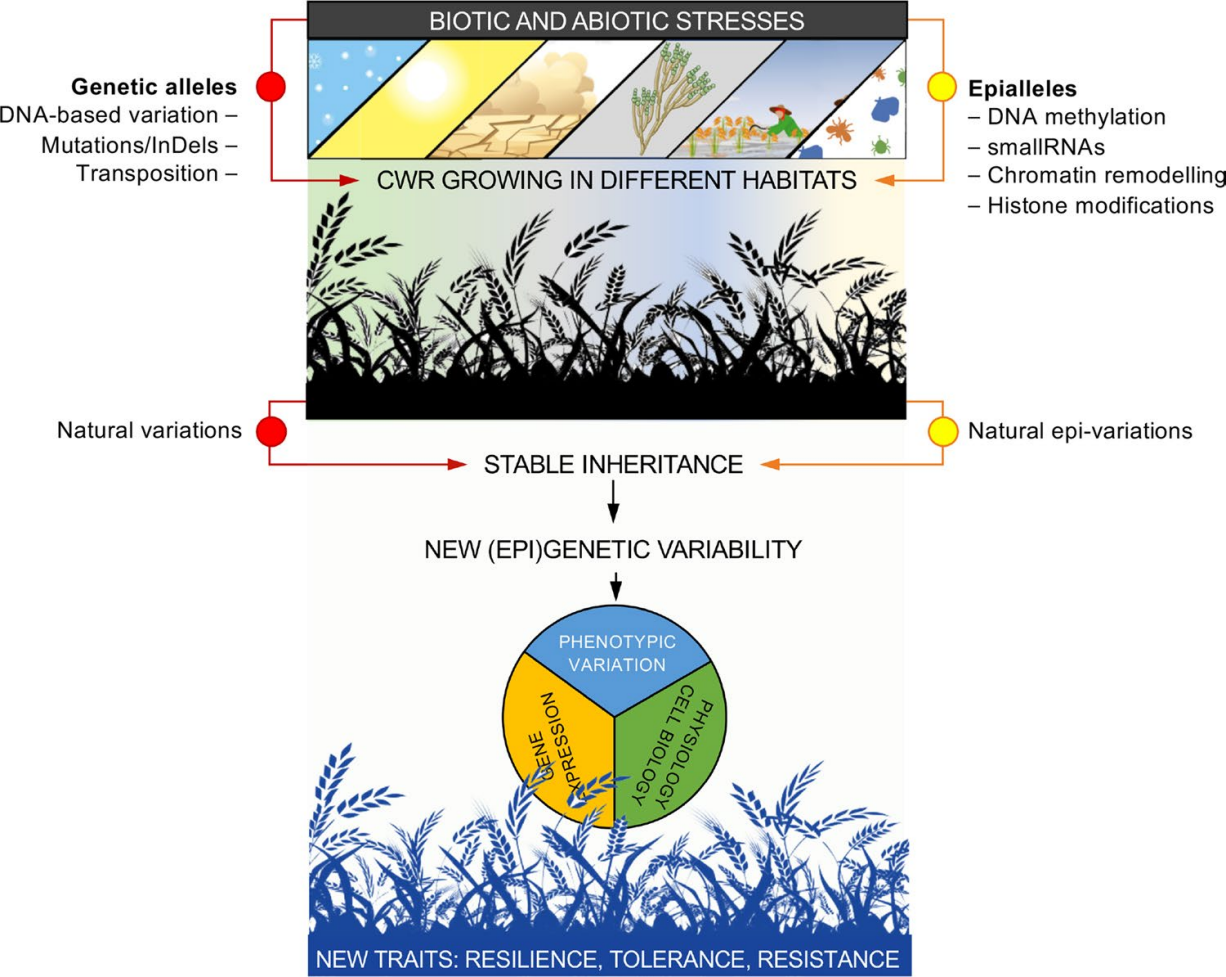
Plant adaptive physiological responses to the environment have long been attributed to variation in genetic and environmental factors which interact and contribute to plant phenotypic plasticity, that is the capacity of a genotype to produce different phenotypes under different environmental conditions. More recently, epigenetic mechanisms have been recognized as component that can both mediate the interaction between genetic and environmental factors and foster plant rapid phenotypic variation under environmental change

Plants are sessile organisms constantly exposed to diverse environments. Many plants developmental processes are greatly influenced by environmental factors, such as day length, light intensity and quality, temperature, and water availability. Environmental factors induced signals are integrated with plant growth and differentiation signals, while contributing to adaptation to different climates (Xiao et al 2017; Fig. 1). Furthermore, environmental factors often induce changes at chromatin level at responsive gene loci and, in some cases, the environment-induced chromatin changes can be transmitted through cell mitosis/meiosis and be memorized by the plants, once the original inducing signal is absent. Particularly, environment-triggered chromatin modifications at certain loci that are stable and mitotically and/or meiotically heritable and can be transmitted to the next generation are referred to as epigenetic changes. Others chromatin changes are transitory and regulate transiently gene expression (He and Li 2018). By altering the competence of genetic information to be expressed, the epigenetic components play a major role in plant interaction and adaptation to both non-stressful and stressful environmental conditions (Baulcombe and Dean 2014). Therefore, epigenetic mechanisms play important roles in integrating environmental signals, contribute to adaptation to different environments, and eventually generate epigenetic variation within a species (Kooke et al. 2015).

According to recent epigenetic models, chromatin remodellers, histone modifiers, and DNA methylating/demethylating activities interact with the mediation of both short and long non-coding RNAs (ncRNAs) for regulating adaptation to the environment (Lamke and Bäurle 2017). They can alter both chromatin states and gene expression and therefore generate plant plastic responses under different environmental conditions. Additionally, they can respond to environmental cues in regulating plant responses to important adaptive processes, such as seed development, vernalization, flowering time, stress responses, and species hybridization (Ahmad et al. 2010) Furthermore, environmental adaptation requires a fine-tuning between the external cues and the timing of plant developmental changes and because often a certain delay occurs between the environmental trigger and the initiation of a differentiation process, a memory of the trigger can be epigenetically set and reset in each generation (Avramova 2015). Intriguingly, one of the earliest characterized processes involving environmental adaptation and epigenetic regulation in plants is vernalization. During vernalization processes, plant cells can record the periods of prolonged cold they have experienced in winter, for flowering in spring. In Arabidopsis, both cold exposure and synchronization of flowering are achieved through a dynamic modification of chromatin properties and consequently of transcriptional state of *FLOWERING LOCUS C* (FLC) transcription factor, the main repressor of flowering. However, the cold-induced epigenetic changes at AtFLC chromatin are reset during gametogenesis to guarantee that the cold exposure and vernalization response synchronize flowering to the optimal season in the following generation (Berry and Dean 2015). Studies of the vernalization process at the mechanistic level in *Arabidopsis thaliana* accessions have demonstrated that non-coding transcription and chromatin regulation influence the expression of the master regulator of flowering *AtFLC* in response to cold. In addition, it has been shown that variations in these mechanisms can favour adaptation in natural populations. These observations confirm that an altered epigenetic regulation, sustained by genetic variation, has the potential to guide adaptation to new and changing environments (Whittaker and Dean 2017).

In plants, other environmental cues and/or stresses appear to be effective at inducing epigenetic changes, which response persists for an extended period after the initial induction, commonly called as a “memory” mechanism that can be adopted in responses to recurring stresses. However, there is relatively strong evidence that these “environmental memories” last a short time and only a few of them show transgenerational features (Sani et al. 2013; Avramova 2015; Martinez-Medina et al. 2016; Forestan et al. 2020). Chromatin marks that are induced by diverse environmental challenges and enriched at genomic loci with regulatory function are often depleted at a later developmental stage or in offspring because this is essential to a regular plant growth and development (Baroux et al. 2011). Although many mechanistic studies of stress effects have been performed for investigating the stress response at the genetic and physiological levels, only more recently epigenetic factors have been taken under consideration, supposing that stress effects on the chromatin level could allow both permanent changes of gene expression and potentially long-term adaptation. For these reasons, many questions are still open on how diverse environmental signals induce chromatin changes at responsive genes and why only certain chromatin marks at specific genomic regions can be transmitted stably to generate somatic or transgenerational environmental memory.

### The heritable epimutations

The term epiallele defines a genetic locus having specific DNA or histone epigenetic modifications that can be stably transmitted to the progeny (Taudt et al. 2016). Epialleles can add a new layer of heritable variation to natural genetic diversity present in the germplasm. The impact of epigenetic modifications on plant phenotypes became apparent two decades ago in a study showing that the naturally occurring morphological variant of a *Linaria vulgaris* was caused by epi-allelic variants in gene *CYCLOIDEA* rather than DNA mutations (Cubas et al. 1999). The same study demonstrated that restoring the original methylation state recovered the wild-type-like phenotype. Since then, it has become clear, by finding new examples of epialleles, that epigenetics played a notable role in plant domestication and evolution (e.g. Manning et al. 2006; Johannes et al. 2009; Olsen et al. 2013; Jiang et al. 2014; Jordan et al. 2016; Rigal et al. 2016; Zhang et al. 2020; Table 1). Although how different epialleles originate at the molecular level in plant population has not been completely clarified yet, it is presumed that they are mainly generated by stochastic spontaneous variations in DNA methylation and/or histone modifications, although the latter case is less documented. A general distinction is made between the so-called pure epialleles generated from non-genetic sources and those which originate as a consequence of underlying genetic variations (Taudt et al. 2016; Hollister et al. 2009). Non-genetic epialleles can originate from spontaneous epimutation due to the failure to perpetuate existing DNA methylation states, as documented in soybean (Shen et al. 2018), maize (Xu et al. 2019), and tomato (Table 1). Additionally, developmental factors or environmental cues that trigger either chromatin mark changes or favour instability of epigenetic states could be sources of non-genetic epigenetic variation. Rearrangements of genome structure and/or transposon insertions close to a gene can cause epigenetic instability and be sources of novel epiallele formation (Pecinka et al. 2013). In particular, the insertion of transposable elements in intergenic regions and their subsequent inactivation through DNA methylation can cause increased methylation around the insertion site and promote the establishment of novel epialleles, as reported in Arabidopsis (Schmitz et al. 2013), rice (Zhang et al. 2015), and maize (Banks et al 1988).

**Table 1:** Breeding value of naturally occurring epialleles in some crop species

Species	Gene/locus	Phenotype	Breeding value	References
Cotton	*COL2D* (CO-LIKE2D)	Loss of photoperiod sensitivity	Positive/improved adaptability, wider growing area	Song et al. (2017)
Maize	*r1* (red 1)	Reduced pigmentation	Positive/genotypes with different seed colour	Brink (1956)
	*b1* (booster 1)	Reduced pigmentation	Positive/genotypes with different seed colour	Patterson et al. (1993)
	*pl1* (purple plant 1)	Reduced pigmentation	Positive/genotypes with different seed colour	Holick et al. (1995)
	*p1* (pericarp colour 1)	Reduced pigmentation	Positive/genotypes with different seed colour	Cocciolone et al. (2001)
	*lpa1 *(low phytic acid 1)	High inorganic phosphate in seed	Positive/improved nutritional quality	Pilu et al. (2009)
	*Spm (*suppressor–mutator)	Anthocyanin production	Positive/improved nutritional quality	Banks et al. (1988)
Melon	*CmWIP1* (WASP/N-WASP-interacting protein 1)	Only female flowers	Positive/facilitation of hybrid production	Martin et al. (2009)
Oil palm	*DEF1* (DEFICIENS)	Mantled fruit	Negative/decreased yield	Ong-Abdullah et al. (2015)
Rice	*D1* (Drawf1)	Dwarf	Positive/facilitation of fruit harvest	Miura et al. (2009)
	*SPL14* (Squamosa promoter binding protein-like)	Panicle branching and higher grain yield	Positive/increased yield	Miura et al. (2010)
	*FIE1* (Fertilization-independent endosperm 1)	Dwarf	Positive/increased yield, easier harvest	Zhang et al. (2012)
	*RAV6* [Related to abscisic acid insensitive 3 (ABI3)/viviparous1 (VP1) 6]	Larger lamina inclination and smaller grain size	Positive/increased photosynthetic efficiency and yield	Zhang et al. (2015)
	*AK1* (Adenylate kinase 1)	Defects in photosynthetic capacity	Negative/albino plants and decreased photosynthesis	Wei et al. (2017)
	*ESP* (Epigenetic short panicle)	Short panicle	Negative/lower yield	Luan et al. (2019)
Tomato	*CNR* (Colourless non-ripening)	Skin pigmentation and fruit ripening	Negative/small fruit with a colourless, mealy pericarp	Thompson et al. (1999); Manning et al. (2006)
	*VTE3* (Vitamin E)	Pigment accumulation and vitamin E biosynthesis	Positive/increased nutritive quality	Quadrana et al. (2014)

An underestimated factor playing a role in generating natural epigenetic variation is the so-called facilitated epigenetic variation. In this, a genetic element involved in controlling specific chromatin-related processes becomes mutated or lost, leading to a natural loss of function or a modifier mutant. An example of the well-known case is a loss of CMT3 CHG DNA methyltransferase in several *Brassicaceae* species, including *Brassica rapa* that seems to coincide with the loss of gene body methylation. A broader phylogenetic analysis revealed that multiple factors from the gene body methylation pathway are missing in gymnosperms and evolutionary older plant groups (Bewick et al. 2017). Another example of such natural loss-of-function mutant includes deletion of *VARIANT IN METHYLATION 1* gene in the specific natural accessions of *A. thaliana*, leading to a reduced DNA methylation level (Woo et al. 2007). Speculatively, spontaneous mutations in the key epigenetic players within populations of self-pollinating species may lead to a burst of new transposon insertions and/or epialleles. Such new variants may remain in the population even after the non-functional allele of the specific trans-acting factor has been eliminated by natural selection and further contribute to natural epigenetic variation.

Recently, Johannes and Schmitz (2019) have proposed that the rate of spontaneous epimutations might be dependent on genetic background and thus conditioned by genetic variation. They also suggested considering the rate and spectrum of spontaneous epimutations “as molecular complex traits” that can be mapped in the genome to identify causative genetic loci and pathways of epigenetic instability. Indeed, this approach is of particular interest for a better understanding of environmental-induced variation at the epigenetic and transcriptional levels since using different species and crops it will help to characterize CWRs and exploit their epigenetic diversity as new sources of variation in plant breeding.

### Epigenetic variation and crop plant propagation

To summarize, both spontaneous occurring and/or environmentally induced epialleles can negatively or positively modulate gene expression and affect plant phenotypes. In crop plants, clonal multiplication provides an opportunity to propagate epigenetic variants associated with traits of interest over many generations. However, during gamete development and early embryogenesis, epigenetic imprints are extensively reprogrammed to reset genomic potential and ensure proper development of the subsequent generations (Borges et al. 2021). While once established, DNA methylation is an epigenetic mark that usually results in transgenerational heritable phenotypic variation, heritability is more variable for the different histone modifications (Baulcombe and Dean 2014). For all these reasons, the exploitation of epigenetic variation for crop improvement is more challenging in species reproduced exclusively by seeds rather than in clonally multiplied plants, in which epibreeding seems not to differ substantially from classical breeding. Therefore, characterization of the epigenetic imprints transmitted to the offspring (heritable epialleles) is critical to drive breeding strategies since they are responsible for a significant phenotypic diversity (Grossniklaus et al. 2013). Furthermore, plants can detect certain environmental conditions during somatic growth and this could trigger epigenetic modifications in a cell lineage that generates the germline (Mirouze and Paszkowski 2011). This could provide a source of increased morphological variation in the offspring that might be subject to natural selection, contributing to the origin of novel epialleles. In this context, analysis of the hidden CWR epigenomic landscapes could enable identifying epialleles and provide valuable tools for crop improvement. Ideally, epigenetic diversity might be generated in plants originating from seeds before being asexually propagated, but unfortunately, this is not possible in many crops.

## CWR epigenetic variation: two examples

Epigenetic variation has a key role in shaping agronomically important traits in germplasm used for crop improvement. In the last decades, several studies indicated that alterations of DNA methylation patterns may facilitate ecological adaptation and might constitute a means for organisms to cope with environmental stress (Bonduriansky et al. 2012). It appears that epigenetic modifications play a particularly important role in fluctuating environments compared to DNA sequence variation (Monteiro et al. 2018). Indeed, CWRs have evolved in ecological conditions that were longer and more extreme than those observed in the relatively controlled environments of agricultural production. Therefore, CWRs represent a reservoir of valuable adaptive traits and a predominantly untapped natural reserve due to a lack of appropriate tools, either genetic or epigenetic, to dissect plant phenotypes and their adaptation (Mace et al. 2013). In addition, epigenetic variation has a key role in shaping agronomically important traits in germplasm used for crop improvement.

### Epigenetic variation in wild emmer wheat natural populations

Tetraploid wild emmer wheat (WEW; 2*n* = 4*x* = 28) (*Triticum turgidum* subsp. *dicoccoides*), found in nature as a wild species, is considered as the primary progenitor of domesticated hexaploid bread wheat and tetraploid durum wheat. Since its rediscovery in Israel by Aharon Aaronsohn in 1906 (Aaronsohn 1910), this species has been extensively studied as a potential donor of beneficial traits to domesticated wheat, including disease resistance, drought tolerance, and nutrient and mineral content (Huang et al. 2016; Klymiuk et al. 2019; Krugman et al. 2018 and reference therein). Its importance as a potential donor for future wheat breeding led to its whole-genome sequencing (Avni et al. 2017). WEW is an annual predominantly self-pollinating species distributed in a patchy manner throughout the Fertile Crescent in diverse environmental conditions (Nevo and Beiles 1989). In Israel, over 140 isolated or semi-isolated populations of WEW were found in regions between Mt. Hermon in the north and Mt. Amasa (Judea desert) in the south. Some of these populations cannot be found to date due to massive urban and road development in Israel and are preserved in gene banks (Krugman et al. 2018). Genetic diversity studies in WEW populations collected across Israel and Turkey were assessed with various genetic markers (Nevo and Beiles 1989; Fahima et al. 1999; Li et al. 1999, 2000; Venetsky et al. 2015; Volis et al. 2014), demonstrating wide genetic diversity within and between populations. While different levels of internal structuring were found within populations, clustering above the population level was non-random and not consistent with geographical proximity and found to be partly correlated with ecogeographic variables. Although these studies assessed the structure and extent of the genetic variation, very little is known about the structure and extent of the heritable epigenetic variation. A survey of epigenetic variation among and within five natural populations of WEW was focussed on the variation in DNA methylation and the contribution of transposable elements (TEs) to this variation (Venetsky et al. 2015). TEs are the largest genome component of most plant genomes, e.g. they account for ~ 80% of the bread wheat genome (Charles et al. 2008; Avni et al. 2017; Clavijo et al. 2017; Appels et al. 2018; Wicker et al. 2018). TEs are silenced by epigenetic means, such as DNA methylation, RNA interference, and chromatin modification, and they might be (at least transiently) activated due to weakening or loss of such control during biotic or abiotic stresses (Pecinka et al. 2010; Ito et al. 2011). DNA methylation levels and patterns were examined in 50 genotypes of WEW from five distinct environments, using an AFLP-based epigenetic marker (MSAP) at random genomic CCGG sites, and CCGG sites flanking TEs using transposon methylation display (TMD). No significant variation in methylation levels was detected between populations. However, population-specific methylation patterns were observed both at random sites and around TEs. Furthermore, it was found that the variation of methylation was eco-geographically structured, suggesting that it might be partly determined by climatic and edaphic factors (Venetsky et al. 2015).

These findings suggest that TEs might play a prominent role in creating eco-geographically structured genetic and epigenetic diversity in wild emmer wheat populations, which might strongly influence local adaptation.

### The role of epi-variation in tomato fruit quality

In the last 20 years, the tomato has become a protagonist in studies regarding epigenetic mechanisms in controlling fruit development and particularly ripening, with effects on quality traits, such as colour, flavour, texture, aroma, and nutritional properties. In particular, landmark reports showed how a substantial level of natural variation in *Solanum lycopersicum* is underwritten by epigenetic processes that govern gene expression and phenotype just as strongly as DNA sequence polymorphisms.

Early evidence on locus-specific DNA methylation epialleles was provided by the characterization of the *Colourless non-ripening* (CNR) locus on a naturally occurred variant. In the *Cnr* epimutant, normal fruit ripening was found to be inhibited because of the hypermethylation of a short region located 2.3–2.5 kb upstream of the *LeSPL-CNR* transcriptional start site (Manning et al. 2006; Zhong et al. 2013). Consequently, *CNR* gene expression was strongly reduced, resulting in colourless and mealy textured fruit pericarp. This phenotype was reverted by silencing key DNA methylation genes in *Cnr* fruits (Chen et al. 2015). Interestingly, the authors discovered that the overall DNA methylation levels differed between tomato cultivars in this region.

Epigenetic variation can also affect quantitative trait loci, as in the VITAMIN E DEFECTIVE 3 (*VTE3*), a gene encoding a 2-methyl-6-phytylquinol methyltransferase and influencing pigment accumulation and vitamin E biosynthesis in tomato. Differences in *VTE3* transcriptional activity have been reported in introgression lines (ILs) obtained from crosses between the wild *S. pennellii* and *S. lycopersicum* (cv. M82). Variation in *VTE3* activity was also detected in several Andean tomato landraces, commercial cultivars, and wild species differing in their capacity to accumulate vitamin E (Quadrana et al. 2014). The authors found that such expression diversity correlated with differences in DNA methylation of the *VTE3* promoter regions and the accumulation of matching small RNAs due to the presence of a *SINE* retrotransposon.

Recently, global genome analyses have offered even more substantial evidence about the regulatory role of the epigenetic marks during tomato ripening. Through a whole-genome bisulphite sequencing (WGBS) approach, Zhong et al. (2013) found substantial changes in the distribution of DNA methylation in tomato fruit, consisting of an extensive loss of methylation as the fruit matures. In addition, the treatment of immature fruits with 5-azacytidine, a well-known inhibitor of DNA methyltransferase 1, caused premature ripening supporting a causal relationship between methylation level and the timing of the ripening process. More recently, the *DEMETER-LIKE DNA DEMETHYLASE 2* (*SlDML2*) gene was found to preside over this process. In the *sldml2* mutant lines, fruit ripening was inhibited via hypermethylation with a negative consequence on the expression of well-known ripening-associated transcription factors and genes involved in carotenoid biosynthesis and ethylene biosynthesis and signalling (Liu et al. 2015; Lang et al. 2017).

Post-translational modifications mediate an additional level of ripening regulation. Among them, the H3K27me3 repressive histone mark plays the main role (Lü et al. 2018). It has been extensively profiled in tomato-specific tissue through ChIP-Seq analysis and was strictly associated with key ripening genes, such as the MADS-box transcription factor *RIPENING INHIBITOR* (*RIN*), the fruit-specific ethylene biosynthetic *ACS2* (1-aminocyclopropane-1-carboxylic acid synthase ACC 2), and *ACO* (ACC oxidase) genes, in non-ripening tissues, such as leaf and immature fruit. H3K27me3 was removed from these regions as the fruit matured, suggesting that its decline might be necessary for ripening induction (Lü et al. 2018). A piece of direct genetic evidence linking H3K27me3 and tomato ripening was shown by Liu et al. (2016) and most recently by Li et al. (2020) through functional characterization of genes controlling histone methylation in tomato fruit. MULTICOPY SUPPRESSOR OF IRA1 (SlMSI1), which is an essential subunit of polycomb repressive complexes (PRC2) for the establishment of H3K27me3 in plants, when overexpressed inhibits fruit ripening by silencing *RIN* and other ripening genes (Liu et al. 2016). Instead, the histone demethylase SlJMJ6, a Jumonji C-terminal (JmjC) domain-containing demethylase, promotes fruit ripening by removing H3K27me3 from the chromatin of *RIN* in addition to a large number of ripening-related genes (Li et al. 2020).

The unifying picture emerging is that phenotypic diversity in tomato is higher than expected based on the available genetic variation alone. However, although much has been learned so far, epialleles and epiQTLs associated with quality traits in tomato remain limited to a handful of examples. This emphasizes the need to explore further the epigenetic variation not only in *S. lycopersicum*, but also within the numerous genetic resources available in public repositories, which comprise, among others, thousands of landraces and several wild-related species. Furthermore, it is urgent to increase the epigenetic toolbox in tomato by achieving additional (epi)mutant lines with characterized offspring.

## Tools for the analysis and characterization of CWR epigenetic variation and its exploitation

Overall, identifying the key epialleles involved in plant development and adaptation to the environment, their mode of inheritance and heritability, and their effect on transcription are instrumental for CWR epigenetic variation exploitation. Next-generation sequencing (NGS) techniques are routinely used to analyse (epi)genomes and transcriptomes in many plant species. One clear advantage of NGS techniques for examining genomes is that they can be applied to both model (those with a genome sequenced and functionally annotated) and non-model (without a genome sequenced or with no or limited annotation) plants. In addition, they allow data comparisons within and among populations and species and across environmental scales. The use of multiple NGS methods, such as genome sequencing and RNA sequencing (RNA-seq and small RNA-seq), can be combined with proteomics and metabolomics and may ultimately make characterization of crop plant diversity possible at functional level. This is of course also fundamental to all steps of PGR conservation and particularly for the utilization of CWRs in plant breeding. In recent years, the most commonly used platforms for high throughput in non-model plant species include Illumina/Solexa, 454/Roche, ABI/SOLiD, and Helicos. Additionally, NGS can be used for studying DNA methylation, DNA–protein interactions, chromatin accessibility, and histone modifications genome wide.

To analyse and interpret the data generated with next-generation sequencing (NGS), a plethora of bioinformatics tools and algorithms have been developed. For instance, more than 20,000 bioinformatics tools are listed in the bio.tools (Ison et al. 2016) database and about 470 of them are in the “epigenetics” category. This massive amount of methods and algorithms can be sometimes overwhelming, especially for researchers approaching bioinformatics for the first time. In order to navigate through the numerous available options, multiple reviews and benchmark studies have been published in the last years. For instance, a survey of best practices for RNA-seq data analysis was published by the Conesa Lab in 2016 (Conesa et al. 2016). Similar works can also be found for the analysis of small RNA-seq data (Mehta 2014), DNA methylation sequencing data (Wreczycka et al 2017), chromatin analysis followed by DNA sequencing data (Nakato and Sakata  2021; Steinhauser et al. 2016; Yan et al. 2020), and multi-omics data integration (Jamil et al. 2020).

This section aims to provide an updated concise description of the most used methods and algorithms that are available to study plant epigenomes.

### Tools for DNA methylation studies

DNA methylation is the best described and understood type of epigenetic variation in plants, which occurs in all cytosine sequence contexts: CG, CHG, and CHH (H represents A, T, or C). DNA methylation is highly enriched in heterochromatic transposable elements (TEs) and repetitive DNA sequences, where it plays a prominent role in transcriptional silencing. Densely methylated gene regulatory regions are also associated with transcription repression. By contrast, cytosine methylation within transcribed regions (called gene body methylation) is found in constitutively transcribed genes. The function of this type of intragenic methylation is not well understood (Bewick and Schmitz 2017), although a recent study reported a role in suppressing intragenic antisense transcripts (Choi et al. 2020). Moreover, methylation of intronic TEs and repeats has been shown to affect mRNA processing mechanisms such as alternative splicing and alternative polyadenylation (Zhang et al. 2018). The establishment, maintenance, and removal of cytosine methylation are catalysed by various enzymes targeted by distinct regulatory pathways. De novo DNA methylation is established through the RNA-directed DNA methylation (RdDM) pathway, which requires specialized transcriptional machinery that comprises two plant-specific RNA polymerases—Pol IV and Pol V—small interfering RNAs (siRNAs) and a growing number of accessory proteins. METHYLTRANSFERASE 1 (MET1) maintains CG methylation, whereas CHROMOMETHYLASE 3 (CMT3) catalyses CHG methylation through a mechanism also involving histone H3K9 dimethylation (H3K9me2). Finally, CHH methylation is maintained by DOMAINS REARRANGED METHYLASE 2 (DRM2) or CMT2, depending on the genomic region (Zhang et al. 2018). Active demethylation in plants is initiated by a family of DNA glycosylases, including REPRESSOR OF SILENCING 1 (ROS1), DEMETER (DME), DEMETER-LIKE 2 (DML2), and DML3, which prevent hypermethylation at multiple genomic locations. These enzymes promote demethylation through a base excision repair pathway and remove methylated cytosines irrespective of sequence context (Zhang et al. 2018). DNA methylation patterns can change either because of failure in maintaining methylation after replication or because of active removal by enzymes (Zhang et al. 2018). In *A. thaliana*, it has been reported that CG “germline” epimutations are about five orders of magnitude more frequent than genetic mutations: about 10^−4^ *vs* about 10^−9^ per site per haploid genome per generation (Shahryary et al. 2020).

Numerous strategies for high-throughput detection of DNA cytosine modifications at whole-genome level have been used in plants. However, the most widespread methods with single-base resolution mainly rely on bisulphite conversion (BS) coupled with NGS (BS-seq). Whole-genome bisulphite sequencing (WGBS) permits to distinguish any 5mC (5-methylcytosine) from C (Lister and Ecker 2009; Lister et al. 2008). Modified BS-seq strategies can be adopted depending on the plant species and genome size, especially in non-model organisms with limited genomic resources. Another procedure that can be used for rapid detection of DNA methylation in specific multiple fragments simultaneously is MSRE-PCR. This procedure is based on extensive digestion of genomic DNA with methylation-sensitive restriction enzyme (MSRE) followed by multiplexed PCR amplification of user-defined genes using gene-specific primers (Melnikov et al. 2005). Additionally, methylated DNA immunoprecipitation sequencing (MeDIP-seq) procedures independent of bisulphite DNA conversion which uses monoclonal antibodies against 5mC have been developed (Taiwo et al. 2012) and can be applied for studying DNA methylation genome wide in crop plants. NGS platforms allow the construction of genomic maps of DNA methylation at single-base resolution and the identification of differentially methylated regions (DMRs), namely genomic regions that exhibit a different methylation status between two groups of samples. In plants, studies analysing variability in DNA methylation identify a high number of DMRs. At population level, DMRs can be related to gene expression and phenotypic diversity (Zhang et al. 2020). Particularly in CWR, DNA methylation studies indicate the presence of substantial epigenetic variation between domesticated genotypes and wild relatives (Song et al. 2017). Two recent articles (Omony et al. 2020; Rauluseviciute et al. 2019) provided a complete review of the most used methods for the analysis of DNA methylation data obtained by bisulphite sequencing and other protocols (i.e. MeDIP). Interestingly, most of the pipelines rely on a reference genome that might not be available for non-model species or crop wild relatives. In such cases, specific tools must be used. For example, in the description of the BsRADseq method (Trucchi et al. 2016) the authors describe the creation of a catalogue of loci from unconverted DNA using the popular STACKS tool (Catchen et al. 2013), which is then used as a reference to perform mapping with BISMARK (Krueger et al. 2011). Marconi et al. (2019) used a slightly similar approach where raw reads are collapsed to create a pseudo-reference genome using the pipeline named MCSeEd. Finally, the epiGBS (van Gurp et al. 2016) pipeline generates a de novo reference using only bisulphite-treated samples followed by read mapping with BWA-METH; variant calling is then used to identify C/T or A/G polymorphisms induced by bisulphite conversion. In addition, the number of reference genomes is also rapidly growing for the CWRs due to the fast development of high-throughput sequencing techniques. Very recently, AlphaBeta, a computational method for estimating the precise rate of stochastic changes in DNA methylation events using pedigree-based DNA methylation data as input, was described (Shahryary et al. 2020). AlphaBeta allows to study transgenerationally heritable epimutations in different plant materials, such as in clonal or sexually derived mutation accumulation lines or in long-lived perennials.

### Tools for chromatin modification and accessibility studies

Chromatin organization affects genome replication, transcriptional silencing, and DNA repair and recombination. Recent studies have demonstrated that in Arabidopsis thaliana functional chromatin is important for genome stability, since loss of DNA methylation or defective nucleosome assembly increases sensitivity to genotoxic stress and alters homologous recombination frequencies (Liu et al. 2015). Different chromatin states are important determinant of gene transcriptional regulation that is affected by nucleosome positioning, histone variants, and post-translational modifications of histones (Vergara and Gutierrez 2017). Covalent modifications to histone tails (H2A, H2B, H3, H4, and histone variants) comprise methylation, acetylation, phosphorylation, ubiquitination, sumoylation, and many others. They are dynamically deposited by specific histone modification enzymes that are referred to as writers. Additional specific enzyme complexes can recognize and remove the covalent modifications and are called readers and erasers, respectively (Zhao et al. 2019). Numerous histone modifications and their modifiers and readers have been identified and characterized in eukaryotes, and although histones and their modifications are highly conserved, the possibility of evolutionary divergence in reading histone modification has been highlighted in plants (Fuchs et al. 2006). Histone H3 and H4 acetylation at lysine residue is enriched in actively transcribed gene chromatin, while histone deacetylation is widespread in condensed chromatin. Similarly, tri-methylation of lysine in position 4 of histone H3 (H3K4me3) and H3K36me3 marks are often observed on actively expressed genes, whereas H3K9me2 is present within heterochromatic regions (Feng and Jacobsen 2011). H3K27me3 is a euchromatic repressive histone mark that can memorize gene repression state with a very high degree of tissue specificity both during plant development and response to environmental cues (Rothbart and Strahl 2014). Chromatin modifiers and markers can be studied genome-wide by using chromatin immunoprecipitation (ChIP) followed by high-throughput DNA sequencing (ChIP-Seq) to identify the DNA regions bound by a specific protein or enriched in specifically modified histones in vivo. To assess whether two different proteins or histone modifications are present at the same site in the genome or determine if a protein coincides with a specific histone modification at the same regulatory element, re-ChIP enables sequential chromatin immunoprecipitations to be performed using two different antibodies (Furlan-Magaril et al. 2009). In crop plants, the information generated by ChIP-Seq strongly depends upon the availability and specificity of antibodies used in immunoprecipitation. However, the ChIP data collected have tremendously contributed to the advancement of our understanding of the mechanism of chromatin signatures in regulating gene expression and chromatin compaction in plants. A further limit in ChIP-Seq applications in crops is the need for large amounts of fresh tissue for chromatin preparation that also requires the development of specific protocols for each plant species and the tissues under investigation. For detecting and functionally characterizing chromatin-bound proteins, together with ChIP-Seq, CUT&RUN (cleavage under targets and release using nuclease; Skene and Henikoff 2017) and CUT&TAG (cleavage under targets and tagmentation; Kaya-Okur 2020) have so far been used in semi-model plants, to overcome some of the limitations of ChIP-Seq, such as the requirement of large amount of input and cross-linking during an initial fixation step. However, they still require the development of efficient protocols before they can be widely applied in crop chromatin studies (Klein and Hainer 2020). Although with a still limited number of applications in non-model plant species, other techniques have been developed to examine DNA accessibility and protein localization on chromatin genome-wide. The main techniques for determining DNA accessibility and nucleosome positioning are DNase-Seq, MNase-Seq, FAIRE-Seq, and ATAC-Seq. In DNase-Seq, DNA–protein complexes are treated with DNase l, followed by DNA extraction and sequencing. Sequences bound by regulatory proteins are protected from DNase l digestion. Deep sequencing provides accurate representation of the location of regulatory proteins in the genome (Boyle et al. 2008). In MNase-Seq, micrococcal nuclease (MNase) derived from *Staphylococcus aureus* is used to treat gDNA before extraction and sequencing (Schlesinger et al. 2013). When using formaldehyde-assisted isolation of regulatory elements (FAIRE), DNA–protein complexes are cross-linked briefly in vivo with formaldehyde. The sample is then lysed and sonicated and after extraction DNA is purified and sequenced: sequencing provides information for regions of DNA that are not occupied by histones (Giresi and Lieb 2009). Assay for transposase-accessible chromatin (ATAC) with high-throughput sequencing relied on the hyperactive Tn5 transposase to fragment genomic DNA in vitro and simultaneously add adapters for high-throughput sequencing. The addition of the adapters would mainly take place in open chromatin regions (Buenostro et al. 2013).

Virtually, all ChIP-Seq data analysis methods rely on “peak calling”, which leads to the identification of protein binding sites by looking for “peaks” of abundance of mapped sequenced reads concerning the average coverage of the genome or to a DNA input. A similar approach can also be used for other techniques not based on immunoprecipitation such as ATAC-Seq, DNAse-Seq, or MNAse-Seq (Minnoye et al. 2021). In the plant community, MACS2 (Zhang et al. 2008) is the most used algorithm for peak calling (Chen et al. 2018). However, other tools like SICER (Xu et al. 2014) and HOMER (Heinz et al. 2010) are possible alternatives. More recently, tools have been published to try to improve the performance of peak calling using machine learning and deep learning techniques such as CNN-peaks (Oh et al. 2020), which is based on a convolutional neural network, or AIControl (Hiranuma et al. 2019), which aims at identifying peaks without the necessity for a matched DNA input sample. It must be pointed out that the machine learning and deep learning methods developed so far have been trained using human or mammalian data; as a consequence, their accuracy on plant data sets should be carefully assessed.

### Small RNAs annotation and analysis

Plant endogenous small RNAs (sRNAs) have a role in almost all biological processes, and they are critical in heritable epigenetic variation. RNAs are functionally classified into two principal categories: microRNAs (miRNAs) and short interfering RNAs (siRNAs; Axtell 2013). miRNAs are typically 21–22 nt long, diced by Dicer 1(DCL1) from single-stranded RNA stem-loop precursors derived by MIR genes transcription. They regulate gene expression at post-transcriptional level, by directing mRNA degradation and translational inhibition (Rogers and Chen 2013). siRNAs originate from double-stranded RNA precursors and are categorized in many different subclasses. The main subclass of siRNAs comprises 24 and 21–22 nt siRNAs that participate in the RNA-directed DNA methylation (RdDM) pathway. Twenty-four nt siRNAs navigate the canonical RdDM to the homologous DNA sequences to trigger DNA methylation. These small RNAs are generated in a complex process involving biogenesis by plant-specific PolIV, DICER, and ARGONAUTE enzymes and silencing with the complex of plant-specific PolV, de novo domains rearranged DNA methyltransferases (DRMs), and other proteins (Matzke and Mosher 2014). The de novo established DNA methylation of young transposons and repetitive sequences by the RdDM will serve as a template for the replication-coupled DNA maintenance methylation executed by the plant maintenance DNA methyltransferases MET1, CMT2, and CMT3. Finally, such loci will be compacted and transcriptionally repressed in the form of constitutive heterochromatin that is generally stable over generations in the plant genomes. 21–22 nt siRNAs are derived from Pol II transcripts and are copied by RNA-dependent RNA polymerase 6 (RDR6) into dsRNAs before their processing by DCL proteins. They participate in the noncanonical RdDM pathway for silencing of young TEs, both transcriptionally and post-transcriptionally (Nuthikattu et al. 2013).

After sRNA sequencing, most small RNA studies are focussed on the detection and characterization of miRNAs, siRNA and secondary siRNA, a further class of siRNA in which biogenesis is triggered by a miRNA-directed cleavage of a coding or non-coding transcript (Lunardon et al. 2020). The Tools4miRs database (Lukasik et al. 2016) contains a list of 205 tools for the analysis of small RNAs. Forty-one tools compatible with plant genomes are listed in the sequencing data analysis section with MiRDeep2 (Friedländer et al. 2012), miRanalyzer (Hackenberg et al. 2011), and the UEA sRNA workbench (Stocks et al. 2012) currently being the most cited. ShortStack (Johnson et al. 2016) is a pipeline able to detect and classify secondary siRNA, such as phasiRNAs and ta-siRNAs, whereas algorithms like NASTI-seq (Li et al. 2013a, b) and NATpipe (Yu et al. 2016) can be used to identify nat-siRNAs. Finally, heterochromatin-associated siRNA (hc-siRNA) can be predicted with miRkwood (Guigon et al. 2019) or ShortStack. Prediction of small RNA targets can be performed with tools like psRNATarget (Dai et al. 2018).

### Omics data integration

An integrated view of the plant genome, epigenome, transcriptome, proteome, and metabolome may be a key factor in applying a translational genomics approach to plant breeding (Choi 2019). Data integration can be performed using statistical methods such as generating correlation networks and clustering or mapping metabolic pathways (Jamil et al. 2020). PaintOmics offers a web-based user-friendly interface to visualize multiple omics data types onto KEGG pathway diagrams (Hernández-de-Diego et al. 2018). mixOmics (Rohart et al. 2017) and iClusterPlus (Shen et al. 2009) provide a multi-variate approach to integrate not only omics data but also phenotypic data, helping to identify meaningful associations and candidate genes.

## Prospects and limits of using CWR epigenetic variation in crop improvement and agrobiodiversity preservation

Recent breakthroughs in epigenetic studies provided evidence that epigenetic variants of functional or agronomic importance exist in CWR gene pools, thus providing a rationale for conservation of epigenotypes of importance to crop biology and breeding, as well as agrobiodiversity preservation.

### Crop improvement

Plant breeding conventionally depends on genetic variability available in a species to improve a particular trait in the crop. To improve both resilience and crop security, exploitation of epigenetic variations and/or the manipulation of the epigenome may be an additional breeding strategy. Epigenetic variants in CWR could be an additional and timely source of variability that could be introduced into crops through epigenetic breeding—epibreeding (Gahlaut et al. 2020). Epibreeding itself does not require selection methods different from those used in conventional crop breeding. Conventional approaches based on the introduction of desired traits from CWR into cultivated varieties can be transferred to epibreeding with the obvious differences in terms of epigenetic variant induction, production, and propagation (Latutrie et al. 2019). Selection trials can be assisted by using epigenetic markers, and traits are further stabilized by using vegetative propagation, where possible, as described by Latutrie et al. (2019). However, the fact that CWR epigenetic structure remains largely unknown despite the substantial interest in evaluating epigenetic diversity in non-model organisms living in nature remains one of the limiting factors for the broader use of CWR in epibreeding (Avramidou et al. 2015a, b). Epigenetic characterization of variants at the population level, which is necessary for epibreeding, is also a limiting factor, since these population-level approaches lack precision (Gourcilleau et al. 2019).

The main question regarding enrichment of the crop gene pool by CWR epialleles and the potential limits in the use of epi-variation for epibreeding pertain to the inheritance of epi-variation and its transgenerational stability. If, for instance, the inheritance of DNA methylation is stable and abiding by Mendelian expectations, then epialleles will be faithfully inherited, and novel epialleles will be rare. However, if DNA methylation patterns are unstable, then a rapid formation, or loss of epialleles within populations can be anticipated (Springer and Schmitz 2017). In the former case, epialleles would be in linkage disequilibrium with proximal genetic polymorphisms in the genome. Therefore, differentially methylated regions (DMRs) can be mapped using GWAS or genomic selection. On the other hand, under mostly unfaithful inheritance, such regions would present limited linkage disequilibrium with proximal SNPs and there would be almost no chance of accurately mapping them on the genome (Springer and Schmitz 2017). The presence of stability regarding DNA methylation inheritance across multiple generations is therefore important. One of the most comprehensive pertinent studies used an *A. thaliana* individual to find a population propagated by single-seed descent for 30 generations (Ossowoski et al. 2010). Although there were only ~ 20 SNPs per individual following 30 generations, thousands of differentially methylated cytosines were found. However, there was greater stability of regional methylation levels than individual modifications (Becker et al. 2011; Schmitz et al. 2011; Springer and Schmitz 2017). Similar results have been found in perennial plants, such as cypress where faithful epigenetic inheritance was manifested about 17 times less than faithful Mendelian genetic inheritance (Avramidou et al. 2015a, b) and in larch (Li et al. 2013a, b). In crop species, different results have been reported even for the same species. For instance, generally stable inheritance with rare examples of unexpected patterns has been shown in recombinant inbred populations of maize (Eichten et al. 2013; Li et al. 2014). Still, non-Mendelian epi-inheritance was reported in maize (Zhao et al. 2008), rice (Peng et al. 2013) and soybean (Schmitz et al. 2013). It could be that some DMRs are associated with proximal structural variation and are in general reasonably faithfully inherited in conjunction with the genetic polymorphism. In contrast, others are associated with stochastic modifications and are not faithfully inherited (Springer and Schmitz 2017).

Furthermore, to have an important role in traditional approaches to plant breeding and improvement, DNA methylation would have to exhibit substantial natural variation within CWR and also influence important traits (Springer and Schmitz 2017). To affect plant traits, natural variation of DNA methylation would probably need to alter gene expression levels (Deng et al. 2017; Zhang et al. 2017). It was found that genes that exhibit qualitative expression differences are more likely to be associated with altered DNA methylation levels, as proved by the analysis of epigenetic recombinant inbred line (epiRIL) populations in *A. thaliana* (Johannes et al. 2009; Reinders et al. 2009). The development of epi-RILs in model species such as *Arabidopsis* has enabled accurate genetic analysis of epigenetic variation and mapping of epigenetic quantitative trait loci (epiQTL) (Gahlaut et al. 2020). This quantitative approach to epigenetic variation provides ample opportunities to dissect the role of epigenetic variation in trait regulation, which can eventually be used in crop improvement programmes and to introduce desired traits for CWR. Thus, epigenetic molecular markers combined with epigenome editing tools can further facilitate the introduction and application of epigenetic-based molecular breeding in important crop plants.

### Agrobiodiversity preservation

In the last decades, CWR genetic resources conservation has evolved from conservation of germplasm accessions for maximizing morphological diversity to the incorporation of data related to genetic diversity, so that germplasm collections that maximize evolutionary history in a manageable number of accessions can be constructed. Identifying natural epigenetic variations within those collections and elucidating their role in adaptation to the environment is important for our understanding of the epigenetic basis of plant reaction to climate change (Baduel and Colot 2021). This is further supported by recent study by He et al. (2018) that highlighted the role of a naturally occurring epiallele in local climate adaptation of *Arabidopsis* accessions, and Xu et al. (2020) that suggested a role of methylation variation in adaptive evolution of maize. However, in order to identify beneficial (epi)genetic variations in CWR and implement them successfully in breeding, careful sampling designs should be set in place that consider the ecological and evolutionary properties of the target species (Hübner and Kantar 2021). That is why further attention should be paid to the development of statistical, analytical, and technical tools for effective sampling design, the germplasm characterization, and its use in crop improvement programs.

The emerging evidence that epigenetic variants may exist in CWR gene pools of functional or agronomic importance provides a rationale for conserving epigenotypes of importance (Kitavi et al. 2020). This strengthens the case for immediate conservation action regarding CWR genetic resources that stems from four main considerations: (a) the need for novel germplasm to enrich crop species against a changing environment, (b) the diversified need for more, better, and variable food given human population growth and the changing standard of living in developing countries, (c) the danger that CWR populations face due to climate change and anthropogenic pressure, and (d) the accumulating evidence that CWRs possess a wealth of useful epigenetic variation that can be used in breeding programmes and provide substantial benefits in the long term. Loss of genetic diversity in crops and genetic erosion within CWR germplasm collections and their natural habitats are slow and long-term processes. Hence, the need for effective conservation programmes for CWR both for widening crop gene pools and agrobiodiversity preservation tends to be overlooked. Without operative CWR conservation programmes, supported by different -omics tools, comprising epigenomics, future breeding progress and agrobiodiversity itself will be at risk (Monteiro et al. 2018).

A major means to secure CWR genetic and epigenetic diversity is the designation of protected areas in situ associated with (epi)genetic monitoring. (Epi)genetic monitoring, which is the quantification of temporal changes in population, genetics, and dynamics metrics, constitutes a method with a prognostic value and an important tool for the protection of biodiversity (Aravanopoulos 2016). The monitoring of genetic resources has been recognized in several international agreements and documents. However, it has not thus far been comprehensively implemented in nature and CWR in particular. Nevertheless, genetic monitoring can be readily and directly applied as an early warning system in the temporal evaluation of any CWR species, especially in marginal and vulnerable CWR populations, as highlighted by climate change scenarios.

## Conclusion

In conclusion, there is a crucial need to improve food and fuel supply production efficiency for an ever-growing population in the modern world. Proper use of epigenetic variation from CWR and other gene pools may provide new opportunities for crop improvement and agrobiodiversity preservation. Advances in -omics have provided new insights into the sources and inheritance of epigenetic variation and enabled the efficient introduction of epi-traits from CWR into crops using epigenetic molecular markers epiQTLs. Furthermore, the development of epigenome-editing tools paved the way for monitoring and manipulating crop epigenomes. For efficient use of epigenetic variation for crop improvement and agrobiodiversity preservation, it will be crucial to pursue research aimed at elucidating how to predict stability for epigenetic variants so that we can use epigenetics for the stable improvement in agronomically important traits.
